# Recurrent Lingual Abscess in an Elderly Female With Bulbar Amyotrophic Lateral Sclerosis

**DOI:** 10.7759/cureus.28280

**Published:** 2022-08-22

**Authors:** Alessandro Carotenuto, Bryant Menke, Joshua Jolton, Jayme R Dowdall

**Affiliations:** 1 Otolaryngology - Head and Neck Surgery, University of Nebraska Medical Center, Omaha, USA

**Keywords:** neurodegenerative disease, tongue, sclerotherapy, amyotrophic lateral sclerosis, lingual abscess

## Abstract

A *lingual abscess* is a rare condition that was scarcely described in clinical textbooks. A lingual abscess recurrence is rare and has only been described twice in the literature. Typically, the tongue and oral cavity have multiple intrinsic properties which stave off intralingual infection; however, there may be situations in which these properties are compromised, as demonstrated in oro-motor disability. Lingual abscesses have the potential to develop into catastrophic obstructive airway issues; therefore, early detection and management are paramount. The following is a presentation of an elderly female with Bulbar Amyotrophic Lateral Sclerosis (ALS) treated conservatively for a lingual abscess with recurrence at eleven months post-treatment. Due to her baseline neuromuscular disorder and elevated anesthesia risk, she was treated in the interventional radiology suite with drain placement and Povidone-Iodine sclerotherapy under conscious sedation with excellent results.

## Introduction

A lingual abscess is a rare condition that was scarcely described in clinical textbooks [1-3]. Most literature surrounding this condition has only been described in case reports. In the pre-antibiotic era, the mortality rate of lingual abscesses was 3%, but now it has become exceedingly rare [2]. Even more rare is a lingual abscess recurrence, which has only been described twice in the literature [4,5]. Since lingual abscesses have the potential to develop into catastrophic obstructive airway issues, early detection and management are paramount [1-3,6]. The following is a presentation of an elderly female with Bulbar Amyotrophic Lateral Sclerosis (ALS) treated for recurrent lingual abscesses.

## Case presentation

**Figure 1:**
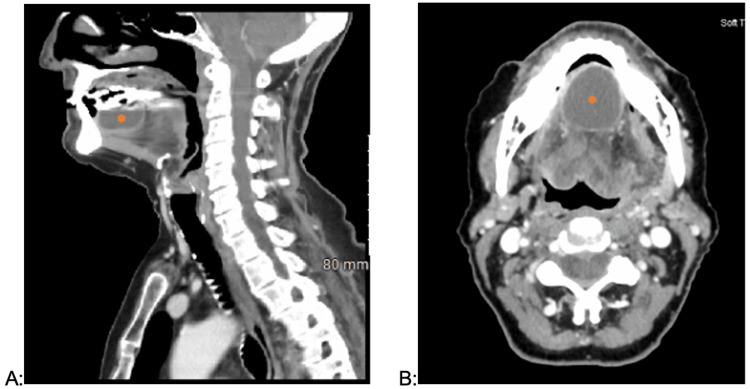
Contrast-enhanced CT imaging of the neck. Contrast-enhanced CT imaging of the neck demonstrated 2.9x3.0x1.5cm peripherally-enhancing fluid collection within the anterior tongue, suggestive of a recurrent lingual abscess. A) Sagittal view, B) Axial View. Asterisk labels lingual abscess.

A 75-year-old female presented to an outside hospital emergency department (ED) with oral pain and tightness of the throat leading to restricted breathing. The patient was presumed to have oral thrush and sent home on anti-fungal therapy. Three days later, she presented with worsening oral pain and tightness causing mildly restrictive breathing and was transferred to our institution for an Otolaryngology consultation. Outside hospital computer tomography (CT) scan demonstrated a 2.9 cm rim-enhancing cystic mass of the anterior tongue contained within the extrinsic musculature (figure 1). The swelling did not violate the submental or submandibular spaces. The presumed diagnosis was a lingual abscess.

The patient has a history of cleft palate repair and bulbar amyotrophic lateral sclerosis (ALS), diagnosed six months prior. She is non-verbal and gastric tube dependent. At baseline, she has dysphagia, dysphonia, and difficulty moving her mouth and tongue. Physical examination of the oral cavity was remarkable for trismus, poor tongue mobility, significant inflammation and edema of the tongue without mucosal changes, and floor of the mouth (FOM) fullness and tenderness to palpation with turbid salivary secretions pooling along the FOM. Indentation was noted upon palpation along the ventral surface of the tongue. The submental and submandibular spaces were tender to palpation as well. The patient was afebrile with a mildly elevated leukocytosis.

A flexible laryngoscopy examination demonstrated a patent and normal-appearing laryngeal airway with narrowing of the oropharyngeal space secondary to posterior displacement of the base of the tongue (BOT) and epiglottis. Needle aspiration resulted in successful decompression and improvement in the patient's perceived breathing obstruction. The aspirate culture showed anaerobic gram-negative rods. She was admitted for airway monitoring and was treated with cefazolin (2000mg, every eight hours, intravenous), metronidazole (500mg, every eight hours, intravenous), and dexamethasone (eight milligrams every eight hours intravenous, three doses). She was discharged three days later and was back to her baseline at follow-up 17 days later.

Eleven months after the initial presentation, the patient presented to the ENT clinic with painless tongue swelling. The patient was promptly transferred to the ED, where a contrast-enhanced CT scan of the neck demonstrated a recurrent lingual abscess. The recurrent tongue abscess was again decompressed via needle aspiration, and she was admitted for further workup of etiology. Cultures this time grew normal oral flora, described as gram-negative rods and gram-positive cocci in pairs, as well as an absence of anaerobic bacteria, fungi, or acid fast bacilli. Blood cultures were negative. She was started on ampicillin-sulbactam (12 grams/day) and dexamethasone. Due to her pre-existing neurologic condition, the decision was made to consult interventional radiology for CT-guided abscess drainage after injection of local anesthesia (1% lidocaine buffered with 8.4% sodium bicarbonate) with the placement of a size six-french drain. Five days later, definitive treatment with Povidone-Iodine sclerotherapy was performed through the existing drain before drain removal. The patient was discharged on hospital day six and returned to baseline health 14 days after discharge.

## Discussion

A lingual abscess is not a prominent feature in head and neck literature, as it is an exceedingly rare problem. Until 2018, it had only been mentioned 85 times in the literature [2]. Because of this, the incidence of lingual abscesses has not been established. It is a problem that very few otolaryngologists will encounter more than once in their careers [3]. The recurrent encounter of a lingual abscess is even rarer, as this has only been described twice in literature (in one adult and a child) [4,5]. While lingual abscesses are rare, they have the potential to create an airway emergency if left unattended.

Predisposing factors related to abscess formation vary by location. For anterior lingual abscesses, predisposing factors include heavy smoking, local trauma, poor dental and salivary health, and, most commonly, impaction of foreign body (fishbone, toothbrush bristles, toothpick, etc.) [1-3,7]. For posterior lingual abscesses, the dominating factors are lingual tonsillitis and infected thyroglossal duct cyst [2,3,7]. The immunocompromised host is at greater risk for infection in either location. To the authors' knowledge, there is no mention in the literature of lingual abscess attributable to craniofacial reconstruction or neuromuscular disorder, as seen in this patient. However, it cannot be sure that her comorbidities did not contribute to recurrence.

The tongue is the dominant structure of the oral cavity and is a frequent disease site. However, deep-seated infections secondary to these various diseases or trauma are rare [3]. Several authors reference various antimicrobial mechanisms inherent to the tongue, including 1) high vascularity and lymphatic supply, 2) tightness of the tongue musculature, 3) integrity of squamous epithelium covering the tongue surface, 4) constant tongue mobility, and 5) continuous turnover of saliva, which is a natural bacteriostatic and lubricating process for the mouth in general [2]. A defect in any of these mechanisms can provide access to a pathologic organism. In the patient described, several of these mechanisms have been impaired due to her baseline oro-motor limitations. These include atrophy and immobility of the tongue, which may result in decreased vascularity to the organ and loss of muscular tension. Additionally, poor salivary clearance results in stagnation and colonization of saliva, potentially compromising the epithelial covering, thus, creating a passageway for microbial seeding. The main pathogens causing lingual abscesses include Streptococcus spp, Staphylococcus spp, gram-negative rods, and anaerobes [1,2].

The most commonly described presentation of lingual abscesses includes odynophagia, dysphagia, painful tongue, poor tongue mobility, and dysarthria [2]. Additionally, fever may or may not be present, depending on the depth and dissemination of the abscess [1]. Most tongue abscesses are unilateral and anterior [6]. Our patient did not present with many of the characteristic features, primarily due to her baseline inability to articulate or swallow. Her two main symptoms were tongue swelling and insidiously worsening respiratory distress. However, her airway complaint was not concerning enough to consider an emergent surgical airway, as might be the case in more acute presentations. The indication for surgical airway is more likely in the case of posterior lingual abscess due to direct oropharyngeal obstruction. However, even anterior or ventral tongue abscesses have the possibility of airway complications in association with epiglottitis and edema [2,8,9]. Left untreated, the natural progression of infection can lead to sepsis or mediastinitis [2].

Diagnosis of lingual abscesses is straightforward, with a thorough history and physical exam comprising the bulk of the workup. However, imaging remains an essential step in evaluation. In 2006, Ozturk presented a series of magnetic resonance imaging (MRI) findings of lingual abscesses and discussed the advantages and disadvantages of various imaging modalities for diagnosing them [6]. CT and MRI are both viable options, each with pertinent advantages and disadvantages. Needle aspiration both confirms the diagnosis and is therapeutic [3,10]. Management of lingual abscesses focuses on three critical items: airway patency, abscess evacuation, and medical management [1-6,10]. The mechanism of airway obstruction is similar to that of Ludwig's Angina, so preparation and close monitoring for emergent surgical airway are paramount, regardless of abscess location [2]. The two most described methods of abscess evacuation are needle aspiration and open surgical drainage. Both can be performed via a transoral or transcervical approach, depending on location and size. The preferred technique is discussed repeatedly in the literature, but there is no clear consensus on which technique is superior. Multiple authors state that needle aspiration is favored in cases where anesthesia is to be avoided to prevent further exacerbation of airway edema via intubation.

Additionally, needle aspiration can be repeated as often as necessary without significant discomfort to the patient [3,5]. Conversely, others advocate for up-front incision and drainage, as needle aspiration alone fails to address the nidus of infection with a higher risk of recurrence, as seen in this patient [1,2]. In the two reports describing recurrent lingual abscesses, both patients were definitively treated with incision and drainage after failed conservative management [4,5]. In the case of a 14-month-old boy, conservative measures failed four times before an open incision in the operating room resulted in definitive treatment [4]. Furthermore, incision and drainage allow tissue collection for histopathology should malignancy be suspected. Depending on location, incision and drainage risk injury to lingual arteries if located in the BOT of FOM [2].

A unique factor in this case report is the pre-existing diagnosis of Bulbar-onset Amyotrophic Lateral Sclerosis. ALS is characterized by the degeneration of upper and lower motor neurons leading to progressive weakness of limbs and the bulbar, thoracic, and abdominal muscles [11]. Onset is divided into three categories, the most common being limb-onset, bulbar-onset, and, rarely, respiratory-onset. Bulbar-onset ALS can be distinguished from an isolated bulbar palsy if the duration from bulbar onset to first significant limb involvement is less than six months [12]. Bulbar-onset accounts for about 30% of ALS initial presentations and affects cortico-bulbar pathways resulting in impaired oro-motor and upper aerodigestive tract function [13]. The patient initially presented to neurology with progressive dysarthria, dysphagia, and emotional lability, followed by signs of progressive upper motor neuron changes in her upper and lower limbs.

Bulbar signs and symptoms pose unique management considerations due to the severe limitations on the upper aerodigestive tract, increased risk of aspiration pneumonia, and early ventilator dependence [11-14]. This significantly affects options for anesthesia. As described in Stoelting's Anesthesia and Co-Existing Disease, general anesthesia in patients with ALS may be associated with exaggerated respiratory depression [14]. With paralysis, they are vulnerable to hyperkalemia secondary to lower motor neuron disease and prolonged responses to non-depolarizing muscle relaxants. Altogether, these risks may ultimately result in tracheostomy placement. General and regional anesthesia are often avoided in favor of epidural anesthesia to spare pulmonary function and symptom exacerbation, where appropriate [14,15]. Thampi et al. describe safely using non-paralytic general anesthesia with regional nerve block for a urologic procedure in a patient with no reported bulbar symptoms but assert that anesthesia in pre-existing neurologic illness must be a nuanced and case-by-case consideration [15]. For these reasons, it was decided to pursue drainage under conscious sedation and local anesthesia in the interventional radiology suite, followed by drain placement and Povidone-Iodine Sclerotherapy.

To the authors' knowledge, this is the first report to describe the definitive treatment of a lingual abscess cavity with sclerotherapy. Sclerotherapy treats various head and neck masses, such as lymphovascular malformations and vascular tumors. It has even been described in treating oral pyogenic granulomas and ranulas [16-18]. Sclerotherapy provides the advantage of obliteration under local anesthesia via injection or a pre-existing drain. Sclerosing agents commonly used include bleomycin, doxycycline, hypertonic saline, Ethibloc (Ethicon/Johnson & Johnson), and sodium tetradecyl sulfate. These agents act to create an inflammatory response and subsequent scarring within the targeted cavity, resulting in obliteration of potential space. Considering the patient's motor deficiencies and already narrowed airway, it was decided not to subject the patient to the possibility of performing a surgical airway if one could be avoided. Given the satisfactory result in this patient, the authors believe sclerotherapy can be considered a safe and reasonable alternative for addressing lingual abscesses in patients when surgical incision and drainage are contraindicated.

Lastly, empiric treatment with broad-spectrum antibiotics should cover the typical offending organisms within the oral cavity (Streptococcus spp, Staphylococcus spp, anaerobic bacteria, and gram-negative rods) [1-10]. In the non-critical patient, oral amoxicillin-clavulanate, clindamycin, and early generation cephalosporin with metronidazole are appropriate regimens [2]. Intravenous antibiotics can be considered for critically-ill patients or if enteral delivery is contraindicated [2].

## Conclusions

A lingual abscess is a rare condition with potentially devastating airway complications left untreated or unrecognized. Several intrinsic antimicrobial properties of the tongue and oral cavity protect against tongue infections. These mechanisms may be compromised in the setting of baseline neuromuscular disability, particularly affecting oro-motor function as seen in Amyotrophic Lateral Sclerosis. Management of a lingual abscess consists of three elements: Airway patency, evacuation of abscess, and antimicrobial therapy. When a general anesthetic is relatively contraindicated in a patient, procedures under conscious sedation or local sedation in the interventional radiology suite may be a viable alternative to the operating room for incision and drainage. One procedure to consider for definitive treatment of a lingual abscess cavity is Povidone-Iodine sclerotherapy.
